# Enterprise Digital Transformation Strategy: The Impact of Digital Platforms

**DOI:** 10.3390/e27030295

**Published:** 2025-03-12

**Authors:** Qiong Huang, Yifan Tang

**Affiliations:** 1 School of International Business, Hunan University of Information Technology, Changsha 410151, China; 2 School of Economics, Hunan Agricultural University, Changsha 410128, China

**Keywords:** digital transformation, digital platform, enterprise strategy, complex network, evolutionary game, information dynamics

## Abstract

The development of the digital economy is a strategic choice for seizing new opportunities in the latest wave of technological revolution and industrial transformation. As a critical tool for driving the digital transformation of enterprises, digital platforms play a pivotal role in this process. This study employs the evolutionary game theory of complex networks to develop a game model for the digital transformation of enterprises and utilizes the Fermi rule from sociophysics to characterize the evolution of enterprise strategies. Throughout this process, the interactive behaviors and strategic choices of enterprises embody the features of information flow and dynamic adjustment within the network. These features are crucial for elucidating the complexity and uncertainty inherent in strategic decision-making. The research findings indicate that digital platforms, through the provision of high-quality services and the implementation of effective pricing strategies, can significantly reduce the costs associated with digital transformation, thereby enhancing operational efficiency and innovation capacity. Moreover, the model reveals the competitive relationships between enterprises and their impact on transformation strategies, offering theoretical insights for policymakers. Based on these findings, the paper proposes policy recommendations such as strengthening infrastructure, implementing differentiated service strategies, and enhancing decision-making capability training, with the aim of supporting the digital transformation of enterprises across various industries and promoting sustainable development.

## 1. Introduction

Amid the rapid development of the global economy, digital transformation has become a crucial pathway for enterprises to enhance their competitiveness and achieve sustainable development [1]. With the rapid advancement of digital technologies such as big data, artificial intelligence, the Internet of Things, and blockchain, enterprises are undergoing profound changes in their operational models, management approaches, and business strategies [2]. Through digital transformation, companies can not only optimize internal processes, increase efficiency, and reduce costs but also create new business opportunities and expand into new markets [3]. In response to this trend, policymakers worldwide are actively promoting digital transformation. On 25 September 2015, at the United Nations Sustainable Development Summit, all 193 UN member states unanimously adopted the Sustainable Development Goals, identifying the development of the digital economy as a key driver of sustainable economic growth, with the aim of achieving 17 Sustainable Development Goals by 2030 [4].

At the heart of corporate digital transformation are digital platforms—comprehensive service systems that integrate advanced digital technologies to provide services such as data sharing, business collaboration, and decision support. These platforms are central to enabling digital transformation by combining technologies like cloud computing, big data, and artificial intelligence [5]. The services offered by digital platforms and their pricing structures play a critical role in shaping enterprise adoption strategies. With the growing range of subscription-based services, enterprises face rising costs, which can deter investment in digital transformation. Alternatively, limited platform services may force enterprises to opt for customized solutions, also increasing transformation costs. Therefore, the quality and pricing of platform services are key factors that influence how enterprises manage the costs of transformation and their overall success in the process.

The role of competition among enterprises further complicates these dynamics. As firms compete to leverage digital technologies for transformation, they must navigate complex decisions around which platform providers to engage with, balancing service quality, pricing, and long-term strategic goals. This competitive environment adds another layer of complexity to the decision-making process, as businesses must optimize their transformation strategies in the face of both technological change and market pressures. Policymakers in regions such as the EU, Japan, South Korea, the United States, and China have recognized the importance of digital platforms in supporting corporate digital transformation, embedding them as key components in national strategies for economic growth [6]. These policies emphasize the pivotal role of digital platforms in driving the digital transformation of enterprises within competitive markets.

Therefore, this paper aims to examine how the service quality and pricing of digital platforms affect enterprise digital transformation, particularly within competitive environments. The goal is to provide both theoretical insights and practical recommendations that support digital transformation efforts. Specifically, the study investigates how digital platform service quality and pricing shape the mechanisms and outcomes of corporate digital transformation, considering the strategic dynamics of competition.

Recognizing that digital transformation is a long-term, dynamic process characterized by gradual strategic evolution, this study incorporates significant network effects arising from the interactions between digital platforms and enterprises. Furthermore, it addresses decision-making complexities—such as uncertainty and bounded rationality—by introducing a complex network evolutionary game model. This model captures the interdependencies and dynamic evolution among enterprises while revealing how network structures and environmental factors influence digital transformation processes. Additionally, the model facilitates an exploration of behavioral evolution and dynamic equilibria under different strategic choices. To enhance the robustness of the analysis, the study uses digital simulation techniques to model enterprise behavior across various scenarios, providing data-driven insights that can guide the optimization of digital platform service quality and pricing strategies. The primary contributions of this research are as follows:

1. It integrates digital platforms and corporate digital transformation within a unified research framework, constructing an evolutionary game model grounded in complex network structures.
2. By examining the information interaction mechanisms and strategic behavioral patterns of enterprises embedded in social networks, the study elucidates the role of digital platforms in the diffusion and emergent processes of corporate digital transformation, enriching the theoretical understanding of digital diffusion.
3. Moving beyond the traditional focus on policy impacts, this research adopts a multi-stakeholder perspective to explore the macro- and micro-mechanisms through which digital platforms influence the diffusion of corporate digital transformation. These findings provide actionable insights for designing effective digital platforms and enhancing the efficiency of corporate digital transformation processes.

## 2. Literature Review

### 2.1. Digital Transformation

Enterprise digital transformation refers to the process of reshaping business models, improving operational efficiency, and fostering innovation capabilities through the adoption of emerging digital technologies [7]. As enterprises undergo transformation, they are better able to absorb and integrate external knowledge based on their accumulated technology and knowledge, thus accelerating the transformation process [8]. However, during this transformation, enterprises often face high costs for digital technologies, and a lack of funding can become a barrier, especially for small- and medium-sized enterprises (SMEs) [9]. Governments can alleviate this financial pressure through policy interventions, particularly fiscal subsidies, which incentivize enterprises to engage in digital transformation and innovation [10].

However, relying on government subsidies is not a sustainable strategy, as it significantly increases the government’s financial burden. Therefore, digital platforms, as important tools for promoting enterprise digital transformation, have become a central focus of research in recent years [11]. Studies show that approximately 80% of SMEs have at least one common priority during their digital transformation, and collaboration among enterprises helps to jointly develop digital technologies and reduce digital risks [12]. As industrial data become a core factor in driving industrial transformation and upgrading, many enterprises are beginning to invest in industrial internet and big data platforms, with business models gradually shifting from consumer internet to industrial internet [13]. Digital platforms help manufacturing enterprises achieve digital transformation by reducing costs, effectively advancing their transformation process [14].

However, due to the diverse and rapidly changing demands during digital transformation, platforms face challenges in dynamically matching these changes, leading to low resource matching and difficulty in meeting personalized needs. Therefore, understanding the commonalities and differences in digital platform services, reducing modular development costs, and improving service quality are essential for more effectively promoting enterprise digital transformation [15,16].

### 2.2. Digital Platforms

Digital platforms play a crucial role in accelerating the digital transformation of enterprises. According to the “Industrial Internet Reference Architecture (v1.9)” released by the Industrial Internet Consortium (IIC) in 2017, digital platforms are pivotal in helping enterprises achieve industrial transformation and enhance productivity [17]. Currently, the research in this field is mainly focused on platform development and technological implementation. For example, Lee et al. evaluated the performance of operating systems for industrial internet applications [18]. In addition, Liu Chao et al. explored the application of industrial IoT technologies in cloud manufacturing systems [19], and Bian et al. studied the implementation and application status of smart IoT platforms for electrical equipment, analyzing the issues faced and the solutions adopted [20]. Zhang et al., based on existing theory and industry practices, proposed a general IIP model, reference architecture, service evaluation system, implementation pathways, and application validation that meet industry needs [21].

However, the service quality and pricing strategies of digital platforms have a profound impact on enterprise participation and the effectiveness of their digital transformation. High-quality services and reasonable pricing are critical in attracting enterprises to engage with these platforms and driving their transformation processes. From a strategic marketing management perspective, the service quality and pricing strategies of digital platforms directly influence whether enterprises choose to adopt these platforms [7]. For small- and medium-sized enterprises, the cost of adopting digital platforms is particularly important. Excessive service costs may deter enterprises from engaging with these platforms, thus hindering their digital transformation efforts [22].

Additionally, the pricing strategy of digital platforms plays a key role in determining the platform’s affordability and accessibility to enterprises. For example, flexible usage-based pricing models or customized pricing for different types of enterprises can significantly impact whether platforms are within reach of companies [22]. Platforms that offer personalized and modular services are often more attractive, as these services can be tailored to the specific needs of individual enterprises, thereby lowering the barriers to digital transformation. However, research in this area is still insufficient, and further exploration is needed on how to optimize pricing and service strategies under different market conditions to facilitate widespread enterprise participation and successful transformation.

### 2.3. Evolutionary Game Theory

Evolutionary game theory (EGT) is an extension of classical game theory that focuses on the evolutionary process of participants (or firms) in long-term strategic interactions within a population. Unlike traditional game theory, which assumes that players are always rational and make decisions based on static utility maximization, EGT emphasizes that, in a dynamic environment, strategy evolution is not only driven by rational decisions but also by irrational behaviors such as imitation, adaptation, and social learning [23]. In EGT, strategies evolve through an ongoing selection process, where more successful strategies gradually dominate the population while less successful strategies are gradually phased out [24].

In the context of digital platforms, EGT can be used to simulate the decision-making process of firms when deciding whether to adopt digital technologies and how to interact with other firms [25]. Firms may choose to cooperate, imitate others, or engage in competition to gain a competitive advantage. The concept of “herd behavior”—where firms tend to follow the actions of others due to uncertainty or lack of information—is particularly relevant in digital transformation, where firms often face uncertainty about the effectiveness of new technologies or strategies.

Moreover, the network structure in which firms are embedded also plays a critical role in digital transformation [26]. Firms’ decisions are not made in isolation but are influenced by the actions of other firms within their network. As firms engage in digital transformation, their decisions are driven by network effects such as cooperation, competition, and social learning, which accelerate or hinder the spread of certain digital technologies and strategies. This creates a feedback loop where early adopters’ decisions influence the choices of other firms within the network, thereby accelerating the diffusion of new technologies and strategies throughout the industry [26,27].

By combining evolutionary game theory with complex network analysis, we can gain a deeper understanding of how firms evolve their strategies within the digital platform ecosystem. This approach not only helps explore how network effects (such as peer influence and social learning) can accelerate or delay digital transformation but also reveals how these dynamics affect firms’ decision-making processes when adopting new technologies [28,29]. Furthermore, the model, combined with numerical simulation technology, has been successfully applied to the study of policy subsidies, corporate competition, and cooperation [30], demonstrating its capability in depicting the evolution of strategies and learning mechanisms.

### 2.4. Mechanisms of Digital Platforms’ Impact on Enterprise Digital Transformation

To gain and maintain competitive advantages, enterprises must continually stay alert to external environmental changes and foster innovation [31]. To achieve this, enterprises should increase their investment in research and development to enhance their digital capabilities, as this is a key strategy for improving the competitiveness of products and services [32]. In the context of the digital economy, empowering enterprises through digital platforms can address issues such as technological deficiencies and funding constraints encountered during digitalization, thereby advancing the digital transformation process [33].

The quality of service provided by digital platforms plays a crucial role in driving enterprise digital transformation. The underlying technologies offered by these platforms, such as cloud computing, big data, and artificial intelligence, enable enterprises to achieve digital transformation at lower costs and higher efficiency [34]. Additionally, the open ecosystems and diverse applications provided by digital platforms allow enterprises to rapidly innovate and transform their business models, continuously optimizing and adjusting their strategies to adapt to the fast-changing market environment [35]. However, incomplete ecosystems and inadequate service offerings may lead to numerous obstacles during the digital transformation process, affecting the overall effectiveness of the transformation [36].

In studying the impact of digital platforms on enterprise digital transformation, it is essential to consider factors such as demand, investment, costs, and pricing. Investment and output are core concerns for enterprise digital transformation, especially regarding service pricing strategies, where the influence of digital platforms is particularly significant [37]. On the one hand, higher service pricing can generate substantial profits for digital platforms, enabling them to further expand and enhance the service quality, thereby providing better digital services to enterprises. On the other hand, higher service pricing increases the cost of digital transformation for enterprises, reducing their demand for digital platform services [22]. High service pricing may become a major barrier to digital transformation, limiting enterprises’ investment and experimentation in this area.

**Figure 1:**
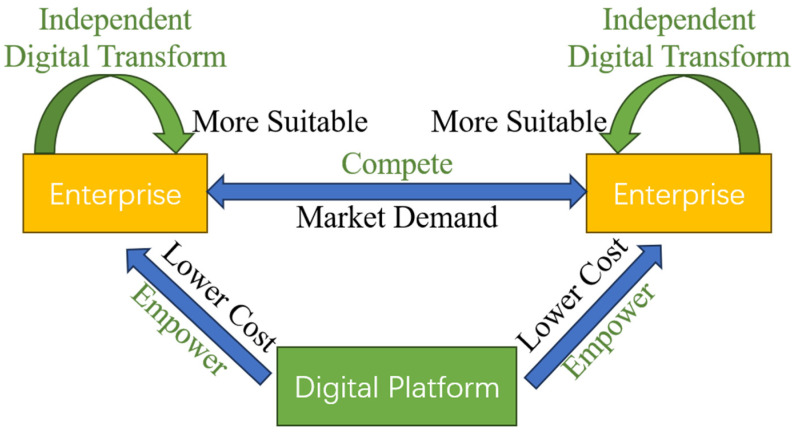
Mechanism analysis.

The network structure between enterprises is a crucial factor in the digital transformation process. Through digital transformation, enterprises can enhance their competitiveness and leverage social network advantages to acquire more potential customers in a constrained market, thus gaining a larger market share from competitors [28]. Additionally, influenced by social networks, enterprises can establish cooperative and communicative relationships with other companies, allowing them to learn from leading firms and drive the advancement and application of digital technologies [29]. Therefore, to effectively advance enterprise digital transformation and overall industry development, it is essential to consider the service quality and pricing strategies of digital platforms, as well as the network structure between enterprises (as shown in Figure 1) [38].

## 3. Model Construction

This study employs complex network-based evolutionary game theory to model enterprise digital transformation, incorporating network structure, game model, and evolutionary rules. The model is designed to analyze how enterprises interact through social networks, learn from each other, and make digital transformation decisions under the influence of digital platforms.

The Newman–Watts (NW) small-world network is used to represent the competitive relationships among enterprises, capturing the clustering characteristics observed in real-world business environments. Enterprises in the network adjust their strategies based on peer interactions, which are influenced by digital platform services, pricing strategies, and technological adoption. The game theoretic framework incorporates strategy evolution and social learning mechanisms, allowing firms to dynamically update their decisions based on market competition and external incentives.

The details of the network structure, game model, and evolutionary rules will be elaborated in the following subsections.

### 3.1. Network Structure

In the same market, different enterprises form a complex social network structure through interactions in various stages such as business, research and development, production, and sales. Therefore, to better align with business practices and systematically explore the impact of digital platforms on enterprise digital transformation, this paper incorporates these complex network relationships into the study of micro-level enterprise decision-making mechanisms. This approach aims to reveal the dynamic evolutionary process of digital transformation within the social network structure. This research is significant for effectively constructing digital platforms.

Complex network models are important tools in the field of mathematics for describing systems composed of many interacting elements. In network science, nodes represent individual elements within the system, and edges represent the interactions between these elements. By analyzing the topological structure of the network, the connectivity characteristics of the nodes, and their dynamic evolution, these models reveal the interdependencies among the various components of the system. There is a wide variety of complex network models, including random networks, small-world networks, and scale-free networks, each with its unique structural features and dynamic behaviors. Among them, the Newman–Watts (NW) small-world network model is widely used to simulate complex systems such as social networks and biological networks due to its characteristics of high clustering and short path lengths [39]. This model can effectively reflect the actual situation of enterprises communicating within smaller social networks, thereby highlighting the clustering characteristics of competitive relationships among enterprises in the real world, providing a new perspective for understanding the propagation mechanism of information in competition and cooperation.

To further explore how digital platforms promote the digital transformation of enterprises, this paper simplifies the network relationships between enterprises to those based on competition. In the same market, enterprises compete for market share through the differentiation of their products and services and learn from enterprises with higher returns based on their social network structure. This learning process is influenced by whether enterprises choose to undergo digital transformation, thereby characterizing the differences in products and services between enterprises. Since the focus of this paper is to analyze the impact of digital platforms on the digital transformation of enterprises, the network structure is handled using the NW small-world algorithm to randomly generate the social network relationships of enterprises. This method not only simplifies the complexity of the model but also makes the observation and analysis of competitive and cooperative relationships between enterprises during the digital transformation process clearer and more systematic.

Let the complex network structure of enterprises be denoted as G=(N,L), where $N=(n_{1},n_{2},\cdots ,n_{m_{1}})$ represents the nodes (enterprises) in the network, and $L=(l_{1},l_{2},\cdots ,l_{m_{2}})$ represents the links between nodes (network relationships of enterprises). For any i,j ∈N two enterprises, if ij∈L (indicating a competitive relationship between the two enterprises), then (i,j)=1; otherwise, (i,j)=0. For any enterprise i∈N, $d_{i}=\sum_{j\in N}\left(i,j\right)$ represents the degree of centrality of enterprise i—that is, the number of enterprises that have a competitive relationship with i.

### 3.2. Game Model

In a competitive market environment, enterprises can adopt three pure strategies: maintaining the status quo without digital transformation (STM), independently undergoing digital transformation (IDT), and leveraging digital platforms for digital transformation (PDT). Enterprises weigh the returns of these three strategies through their social network relationships to determine their final strategy choice. Specifically, maintaining the status quo may avoid short-term risks and costs but may miss market demands; independently undergoing digital transformation can better meet the enterprise’s personalized needs but may face high development and implementation costs; leveraging digital platforms for transformation allows the sharing of platform resources and technology, reducing transformation costs, but may be constrained by the platform’s service quality and pricing. When an enterprise chooses the IDT strategy, it incurs digital transformation costs I, and the unit production cost of its products decreases to $C^{DT}$. When an enterprise chooses the PDT strategy, it pays the digital transformation cost αI to the digital platform, and the unit production cost decreases to $C+\beta \left(C^{DT}-C\right)$, where α and β $\left(0\leq \alpha \leq 1,\;0\leq \beta \leq 1\right)$ represent the platform’s pricing factor and service factor (hereinafter referred to as the pricing factor and service factor), respectively. When α=1, the platform’s charges for digital services are the same as the cost of independent digital transformation. When β=1, the platform’s service quality fully meets the enterprise’s digital needs, identical to the independently achieved digital content. When choosing the STM strategy, the enterprise’s operational costs remain unchanged.

Suppose there are N enterprises in the market, all producing the same homogeneous product. The unit production cost of this product is C, the market price is P, and the total demand is Q. In the initial state (where none of the enterprises have chosen digital transformation), the average market demand faced by each enterprise is $q=\frac{Q}{N}$. For any enterprise i∈N, let the number of enterprises in its network neighbors adopting the STM strategy be $d_{i}^{STM}$, the number adopting the IDT strategy be $d_{i}^{IDT}$, and the number adopting the PDT strategy be $d_{i}^{PDT}$, where $d_{i}=d_{i}^{STM}+d_{i}^{IDT}+d_{i}^{PDT}$. For any digitalized enterprise i∈N (enterprises adopting either the IDT or PDT strategy), if there are traditional enterprises j∈N (enterprises adopting the STM strategy) in the competitive relationship, then enterprise i can acquire a portion of the market share from enterprise j. Assume the acquisition ratio is θ.

**Figure 2:**
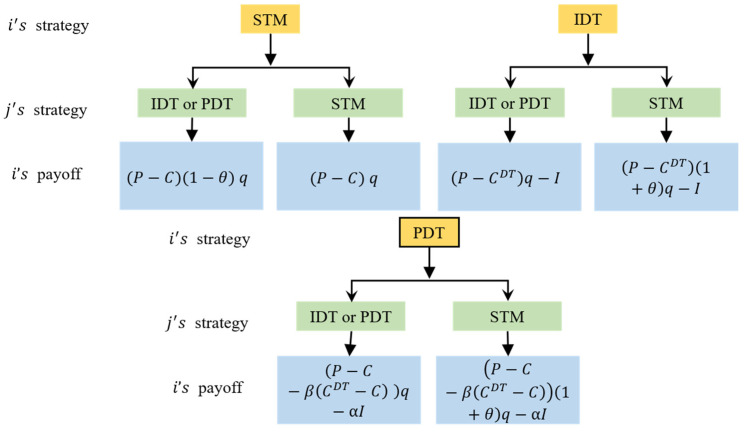
Different payoffs for enterprise *i* under various scenarios.

Based on the above assumptions, take any enterprise i∈N and its competitive enterprise j∈N as an example to construct the payoff matrix for enterprise i (as shown in Figure 2).

In the game model, the payoffs for enterprise i are described as follows.

When enterprise i adopts the STM strategy and enterprise j adopts a digital strategy (IDT or PDT), enterprise j gains a portion of the market share from enterprise i through digital transformation. In this case, the market demand for enterprise i is $\left(1-\theta \right)q$, with the unit product price and unit production cost being P and C, respectively. The profit for enterprise i is (P−C)(1−θ)q.

When both enterprise i and enterprise j adopt the STM strategy, enterprise j does not gain any market share from enterprise i. In this scenario, the market demand for enterprise i is q, with the unit product price and unit production cost being P and C, respectively. The profit for enterprise i is (P−C)q.

When enterprise i adopts the IDT strategy and enterprise j adopts a digital strategy (IDT or PDT), enterprise j does not gain any market share from enterprise i. In this case, the digital transformation cost for enterprise i is I, with the unit product price and unit production cost being P and $C^{DT}$, respectively. The market demand is q, and the profit for enterprise i is $(P-C^{DT})q-I$.

When enterprise i adopts the IDT strategy while enterprise j adopts the STM strategy, enterprise i gains a portion of the market share from enterprise j through digital transformation. In this scenario, the market demand for enterprise i is $\left(1+\theta \right)q$, with the unit product price and unit production cost being P and $C^{DT}$, respectively. The profit for enterprise i is $(P-C^{DT})(1+\theta )q-I$.

Similarly, the following cases can be calculated.

When enterprise i adopts the PDT strategy and enterprise j adopts a digital strategy (IDT or PDT), the profit for enterprise i is $(P-C-\beta (C^{DT}-C))q-\alpha I$.

When enterprise i adopts the PDT strategy and enterprise j adopts the STM strategy, the profit for enterprise i is $\left(P-C-\beta (C^{DT}-C)\right)\left(1+\theta \right)q-\alpha I$.

Based on the above network structure and game model, the expected profit for enterprise i is calculated as follows:

When enterprise i adopts the STM strategy, the expected profit is

$$U_{i}^{STM}=\left(\begin{array}{cc} \frac{d_{i}^{STM}}{d_{i}} & 1-\frac{d_{i}^{STM}}{d_{i}} \end{array}\right)\times \left(\begin{array}{c} \left(P-C\right)\;q\\ \left(P-C\right)\left(1-\theta \right)q \end{array}\right).$$

When enterprise i adopts the IDT strategy, the expected profit is

$$U_{i}^{IDT}=\left(\begin{array}{cc} \frac{d_{i}^{STM}}{d_{i}} & 1-\frac{d_{i}^{STM}}{d_{i}} \end{array}\right)\times \left(\begin{array}{c} \left(P-C^{DT}\right)\left(1+\theta \right)q-I\\ \left(P-C^{DT}\right)q-I \end{array}\right).$$

When enterprise i adopts the PDT strategy, the expected profit is

$$U_{i}^{PDT}=\left(\begin{array}{cc} \frac{d_{i}^{STM}}{d_{i}} & 1-\frac{d_{i}^{STM}}{d_{i}} \end{array}\right)\times \left(\begin{array}{c} \left(P-C-\beta \left(C^{DT}-C\right)\right)\left(1+\theta \right)q-\alpha I\\ \left(P-C-\beta \left(C^{DT}-C\right)\right)q-\alpha I \end{array}\right).$$

### 3.3. Strategy Update Rules

In the real business environment, the bounded rationality of enterprise decision-making and the homogeneous behavior exhibited during digital transformation demand a more nuanced approach to constructing behavioral models for economic agents. These complexities require models capable of capturing the micro-level differences and irrational behaviors in individual decision-making. In this context, the Fermi–Dirac statistical rule, compared to traditional magnetism and statistical mechanics models, demonstrates greater precision. This rule not only captures the complexities of individual decision processes, including interaction effects and the diversity of strategy choices, but also simulates decision-making behavior when economic agents face resource or opportunity constraints.

Thus, within the realm of sociophysics research, scholars increasingly apply the Fermi–Dirac rule when exploring irrational behavior and bounded rationality in decision-making processes [40]. The application of this rule enables researchers to gain a deeper understanding of individual behavioral motivations and patterns in socio-economic systems, thereby providing theoretical support and empirical evidence for the design and optimization of economic strategies.

By analogizing individual strategy choices with the behavior of fermions occupying energy states, this paper adopts the Fermi–Dirac rule as the strategy update rule for enterprises, reflecting the bounded rationality in their strategy selection. In each round of the game, any enterprise i∈N will compare itself with a competing enterprise with a certain probability. If the compared enterprise has a higher payoff, the enterprise will learn from it in hopes of achieving a higher payoff. The expression for this rule is

$$P\left(s_{i}\to s_{j}\right)=\frac{1}{1+e^{\frac{\left(U_{i}-U_{j}\right)}{k}}},$$

where $s_{i}$ and $s_{j}$ represent the strategies chosen by enterprises i and j, respectively, $U_{i}$ and $U_{j}$ represent the expected payoffs obtained by enterprises i and j under their respective strategies $s_{i}$ and $s_{j}$, and parameter k represents the level of irrationality of enterprise i, which can amplify or diminish the enterprise’s perception of payoff differences. This expression indicates that, as the payoff difference between enterprise i and enterprise j increases, the probability of enterprise i adopting the strategy of enterprise j also increases, and this probability rises as the level of irrationality k decreases.

In addition, recognizing the critical interplay between information flow among enterprises and the evolution of decision-making during the game process, information asymmetry and bounded rationality are identified as key factors in corporate decision-making. The efficiency and methods of information dissemination exert a direct influence on the evolution of enterprise behavior. However, the traditional application of the Fermi–Dirac rule in social contexts does not adequately address the issue of information asymmetry, wherein enterprises cannot immediately access the revenue data of all competitors. To better reflect real-world dynamics, this study assumes that, in each round of the game, enterprises probabilistically select a single competitor and update their strategies according to the Fermi–Dirac rule. This adjustment accounts for the partial and probabilistic nature of information acquisition in practical settings, thereby improving the model’s alignment with real-world conditions and enhancing its applicability and theoretical robustness.

After the model experiences a certain period of evolution, the number of enterprises choosing digital transformation will reach a progressively stable level. Let $N^{STM},N^{IDT},$ and $N^{PDT}$ represent the sets of enterprises adopting the STM, IDT, and PDT strategies, respectively. The number of enterprises choosing the STM, IDT, and PDT strategies are denoted by $\left|N^{STM}\right|,\;\left|N^{IDT}\right|$, and $\left|N^{PDT}\right|$, respectively. The average payoffs for non-digital and digital enterprises are represented by $\bar{U_{IDT}}=\frac{\sum_{i\in N^{IDT}}U_{i}}{\left|N^{IDT}\right|}$ and $\bar{U_{PDT}}=\frac{\sum_{i\in N^{PDT}}U_{i}}{\left|N^{PDT}\right|}$, respectively. The digitalization level of the industry is denoted by $\frac{\left|N^{IDT}\right|+\left|N^{PDT}\right|}{N}$.

## 4. Simulation and Analysis

### 4.1. Simulation Steps and Initial Settings

Based on the above model settings and existing research paradigms, this study uses MATLAB (R2024b) to analyze the evolution and results of enterprise digital transformation in detail. The specific steps are as follows.

**Figure 3:**
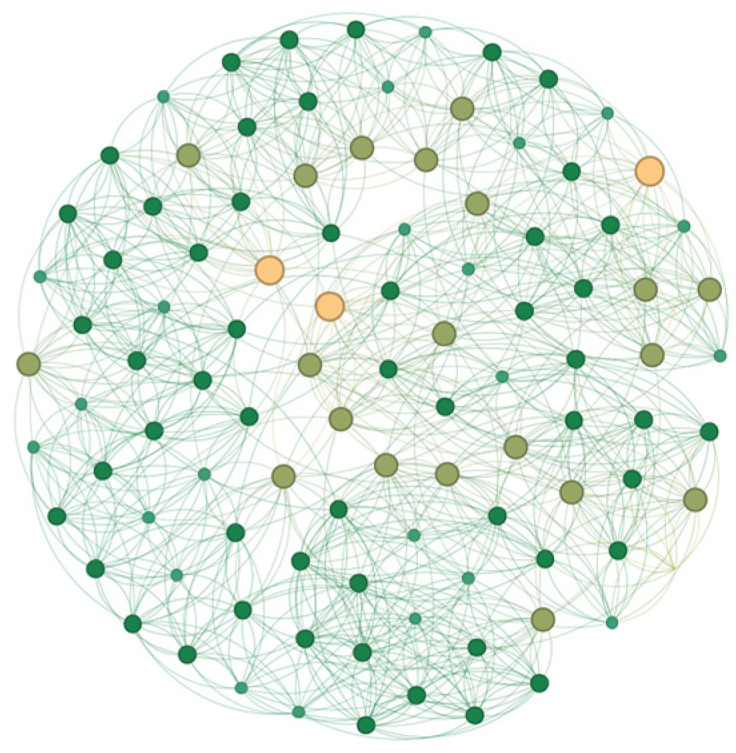
NW network structure.

(1) Create a NW small-world network containing enterprises N, with the generation process divided into two main parts. First, construct a regular ring network where each enterprise is connected to an average of the nearest neighbors ω. Specifically, each node forms a closed circular structure with its adjacent left and right nodes. Next, introduce small-world characteristics by randomly reconnecting some edges. This process is achieved by setting a reconnection probability p (here, p=0.3), allowing nodes in a sparse network to quickly transmit information through a few intermediary nodes (as shown in Figure 3). Additionally, set the initial proportion of digital enterprises among the enterprises N according to the ratio γ.

(2) Enterprises engage in evolutionary games within the NW small-world network. Based on the game model and network structure, calculate the payoff for each enterprise. Each enterprise randomly selects a competing enterprise for comparison and calculates the probability of learning from that enterprise based on the Fermi rule. Subsequently, enterprises learn digital transformation strategies from competing firms according to this probability, thereby optimizing their decisions and enhancing market competitiveness.

(3) Repeat the evolutionary game process until the system reaches a stable state. During each iteration, enterprises continuously update their strategies based on strategy adjustments and payoff outcomes. The system is considered to have reached a stable state when the strategies of enterprises no longer undergo significant changes or meet the established convergence criteria.

(4) Perform multiple simulations to reduce errors. The entire process from Steps 1 to 3 is repeated 20 times. By averaging the results of each simulation, random process-related errors can be effectively minimized, thereby improving the accuracy and representativeness of the final outcomes.

To thoroughly analyze the role of digital platforms in enterprise digital transformation, this study employs simulation analysis to conduct detailed simulations of pricing and service factors under different scenarios. During the construction of the simulation model, key parameters were selected based on data from industry reports and academic literature to ensure the generalizability of the research results and the applicability of the theoretical framework. The initial parameter settings are shown in Table 1.

In terms of simulation parameter selection, factors such as the number of enterprises N, the number of neighboring enterprises ω, and the initial proportion of digitalized enterprises γ are based on existing research to ensure comparability and consistency of the results [41]. For specific market scenarios of digital platform services, this study adjusts certain parameters to more accurately reflect the market characteristics of the platform’s target clients. Market demand Q is set at 2.5 million, a figure derived from in-depth industry analysis that reflects the market demand faced by enterprises undergoing digital transformation via digital platforms. This setting considers the relationship between market demand and the motivation for digital transformation. High market demand may drive enterprises to undertake digital transformation independently, rather than relying on a platform.

The digitalization cost I is set at 550,000 (RMB), based on an analysis of the costs for enterprises to undergo independent digital transformation. This reflects the potential of digital platforms to offer cost-effective solutions that help enterprises achieve cost savings. Additionally, considering that personalized demands studied in the existing literature may lead to higher production costs and product prices, this study adjusts production cost and product price accordingly to better simulate the actual conditions of platform services for enterprises.

The post-transformation production cost $C^{DT}$ is set at 80, aiming to reflect the cost-efficiency achieved through digital transformation. The service quality and pricing factors, as core parameters of this study, are assigned an initial value of 0.5 after considering both the simulation analysis and real-world conditions. This setting is intended to simulate the wide applicability and cost-effectiveness of digital platform services for enterprises while avoiding extreme values that could skew the results.

### 4.2. The Impact of Digital Platforms on Enterprise Digital Transformation

**Figure 4:**
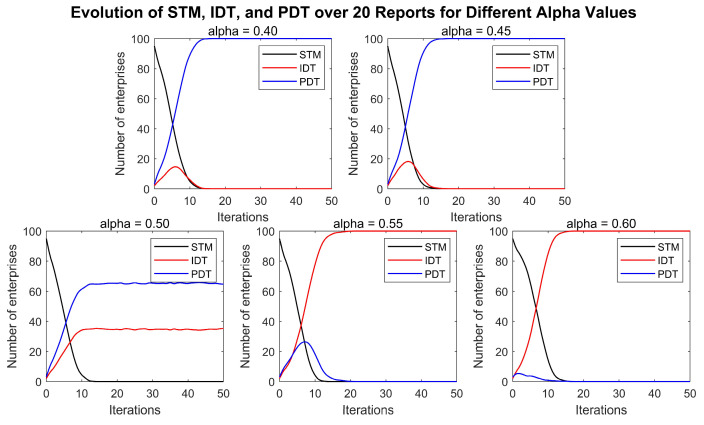
The impact of the digital platform pricing factor on enterprise digital transformation.

Figure 4 shows the simulation results of the impact of the pricing factor on enterprise digital transformation. The figure illustrates the effects of different platform pricing on digital transformation when the service content fit of the digital platform is β=50% and the pricing factor α takes the values of 0.4, 0.45, 0.5, 0.55, and 0.6, with all the other conditions remaining constant.

After 50 rounds of the game, it was observed that, under specific market conditions, digital transformation is an inevitable choice for enterprises to enhance their market competitiveness. Regardless of the pricing set by digital platforms, all enterprises ultimately opt for digital transformation. Additionally, the results indicate that, when the quality and pricing of digital platform services are each at half the level of independent digital transformation, the ratio of enterprises choosing the IDT strategy to those choosing the PDT strategy is approximately 4:6. When the service quality of the digital platform remains unchanged, a lower platform pricing leads all enterprises to adopt the PDT strategy for transformation. However, as the pricing of digital platform services increases, the number of enterprises opting for the IDT strategy gradually rises.

This outcome may be due to the fact that, in the long term, digital transformation significantly enhances production efficiency and market share. Despite the initially high costs of digital transformation, the subsequent savings in production costs and increased market demand are sufficient to offset these drawbacks. Low-cost, high-efficiency digital platform services are highly attractive to enterprises, because they significantly reduce the cost of transformation. When digital platform services offer high cost-effectiveness, enterprises are more inclined to use PDT, as this reduces the complexity and risks associated with digital transformation. However, some enterprises may choose the IDT strategy to gain greater autonomy and customized services during independent transformation.

Nevertheless, as the pricing of digital platform services rises, more enterprises opt for the IDT strategy. This shift may be due to the high service fees prompting enterprises to consider utilizing their own resources or seeking alternative solutions for digital transformation, thus avoiding the high costs of platform services. The diversity in strategy choices reflects rational decision-making by enterprises in response to varying market conditions and cost structures.

**Figure 5:**
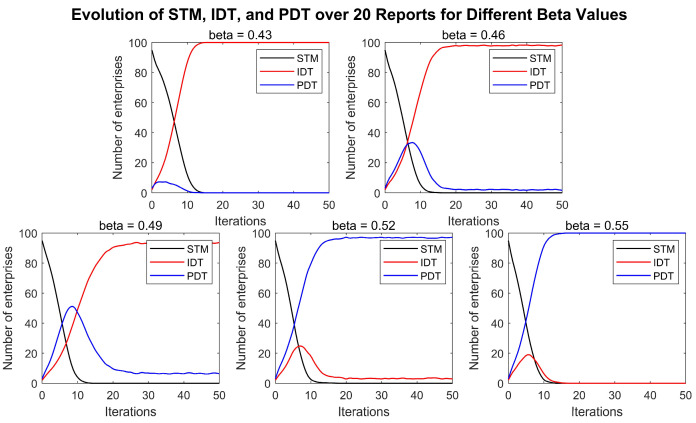
The impact of the digital platform service factor on enterprise digital transformation.

The simulation results of the impact of the service factor on enterprise digital transformation are shown in Figure 5. Figure 5 illustrates the effect of different service factors on enterprise digital transformation when the service pricing of the digital platform is fixed. The service factor is varied at the values of 0.43, 0.46, 0.49, 0.52, and 0.55, while the other conditions remain unchanged.

Similarly, after 50 rounds of the game, it was found that, regardless of the service quality provided by the digital platform, all enterprises eventually opted for digital transformation. The results further indicate that, with other conditions held constant, if the cost-effectiveness of the services provided by the digital platform is lower than that of the independent digital transformation (i.e., the platform’s service cost is 50% of the independent transformation cost, but the quality is less than 50% of that of independent transformation), nearly all enterprises will choose the IDT (independent digital transformation) strategy. As the range of services provided by the digital platform becomes more comprehensive and its cost-effectiveness improves, enterprises will increasingly prefer to rely on the platform for their transformation, ultimately choosing the PDT (platform-dependent transformation) strategy for digital transformation. This outcome is likely due to the fact that, when the cost-effectiveness of the digital platform is lower than that of independent digital transformation, relying on the platform does not yield sufficient benefits, leading enterprises to prefer independent digital transformation. However, as the digital platform’s service offerings become more robust, enterprises find that using the platform enhances the efficiency of the transformations, making them more willing to rely on the platform. Although enterprises initially adopt various strategies for digital transformation, since most enterprises have not yet undergone digital transformation at the outset, any enterprise that does transform can reduce production costs and gain a market share. As the number of digital enterprises increases, firms become more cautious in choosing digital strategies. At this stage, with most competitors also having undergone digital transformation, the potential market share gains from transformation become limited, thus making enterprises more inclined to select strategies with higher cost-effectiveness.

Table 2 shows the average payoffs for enterprises choosing the IDT and PDT strategies under different combinations of pricing and service factors in the steady state of digital transformation. As the pricing factor α increases in increments of 0.05 within the range [0.4–0.6], the number of enterprises opting for the PDT strategy gradually decreases, and their average payoffs also decline, while the number of enterprises choosing the IDT strategy increases. In contrast, when the service factor β increases in increments of 0.03 within the range [0.43–0.55], the number of enterprises adopting the PDT strategy gradually increases, and their average payoffs rise accordingly, while the number of enterprises choosing the IDT strategy decreases.

Additionally, to further reveal the role of digital platform service pricing and service quality in the micro-level enterprise decision-making interaction mechanism, a comparative analysis of the average revenue of enterprises undergoing digital transformation is conducted.

**Figure 6:**
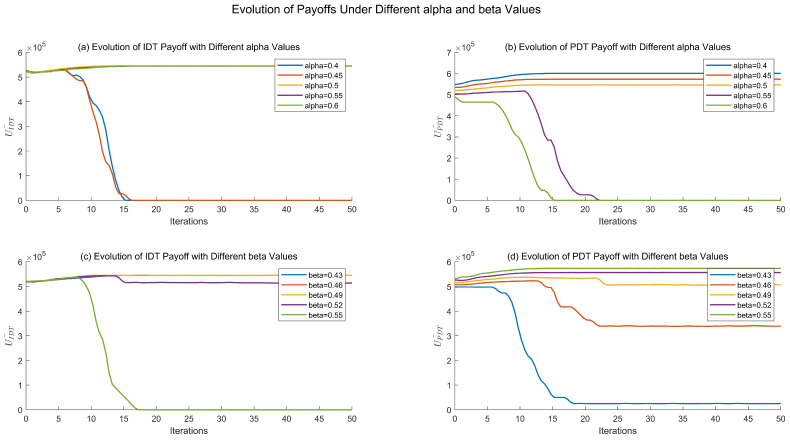
Evolution of payoffs under different α and β values.

Figure 6 illustrates the changes in average revenue for enterprises under different digitalization strategies, as service pricing and service quality vary. From Figure 6a,b, it can be observed that, when α=0.5, $\bar{U_{IDT}}$ and $\bar{U_{PDT}}$ are approximately equal. Regardless of the value, the average revenue of enterprises choosing IDT and PDT strategies is similar at the initial state. However, over time, when α<0.5, the average revenue of enterprises adopting the IDT strategy for digital transformation gradually decreases. Conversely, when α>0.5, the average revenue of enterprises opting for the PDT strategy gradually declines.

This trend can be attributed to the high importance enterprises place on the return on investments for digital transformation. When α=0.5, the cost-effectiveness of the two strategies is comparable, resulting in a similar number of enterprises choosing either strategy and, thus, similar average revenues. In the initial stages, since market competition is not yet intense, the difference in revenue between enterprises adopting IDT and PDT strategies mainly reflects the differences in digital transformation costs and product production costs. Digital transformation compensates for this difference by expanding market share, so regardless of the value of α, the average revenues for both strategies are similar in the initial stage. As the number of digital enterprises increases, the ability of enterprises to gain market share through digital transformation gradually diminishes, leading to increased sensitivity to digital platform pricing. Consequently, when α>0.5, the number of enterprises choosing IDT will increase to 100%, eventually leading to a reduction in the number of enterprises adopting PDT to zero, with the average revenue also dropping to zero. Conversely, when α<0.5, the number of enterprises opting for PDT will increase to 100%, causing the number of enterprises choosing IDT to drop to zero, with the average revenue also falling to zero.

Similarly, Figure 6c,d show that, as the range of services provided by the digital platform increases, enterprises are more inclined to use the platform for transformation, aiming for higher average revenues. Combining this with the data from Table 2, it is evident that both the service quality and service pricing of the digital platform have a comparable impact on enterprise digital transformation.

### 4.3. Sensitivity Analysis

#### 4.3.1. Initial Proportion

**Figure 7:**
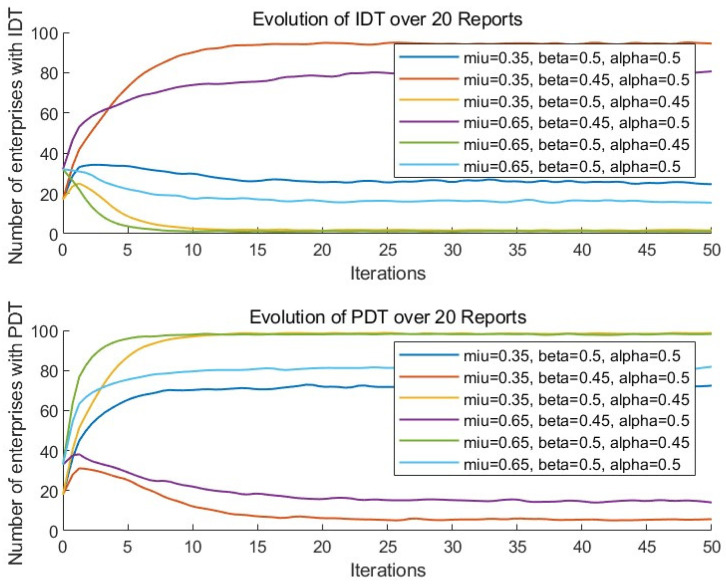
Sensitivity analysis of different initial proportions of digital enterprises.

To examine the impact of different initial proportions of digital enterprises γ on the evolution of enterprise digital transformation, simulations were conducted with γ set to 0.35 and 0.65. The results are shown in Figure 7. It can be observed that, as the initial proportion of digital enterprises γ increases, the conclusions regarding the impact of digital platform pricing and service quality on enterprise digital transformation remain valid. Additionally, under constant conditions, as the initial proportion of digital enterprises increases, the number of enterprises choosing the IDT strategy for digital transformation decreases, while the number of enterprises adopting the PDT strategy gradually increases. This indicates that the choice of digital transformation strategy is related to the initial proportion of digital enterprises. When the initial number of digital enterprises is high, enterprises are more likely to choose the PDT strategy for digital transformation and are more sensitive to the pricing and service quality of the digital platform.

#### 4.3.2. Network Size

**Figure 8:**
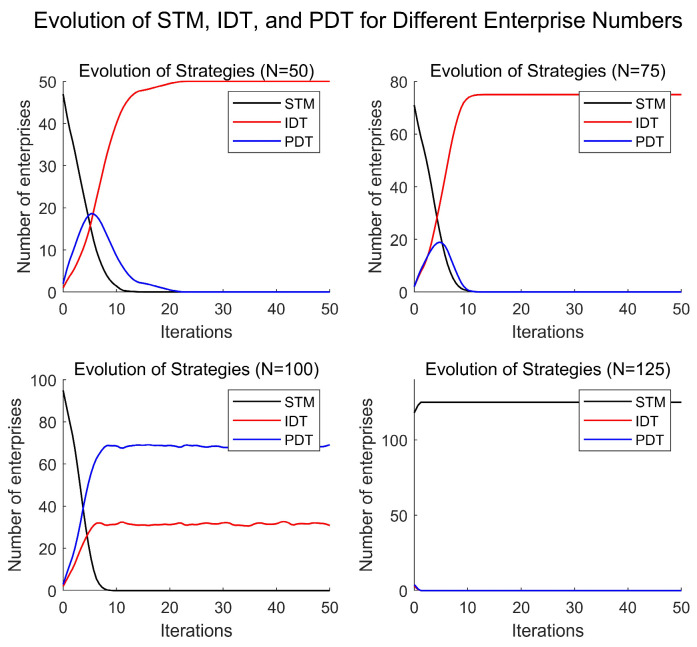
Enterprise digital transformation under different network scales.

To assess the impact of network size N on the conclusions, numerical simulations were conducted with network sizes of 50, 75, 100, and 125. The results are illustrated in Figure 8. By comparing the curves for different network sizes, it can be observed that, under the same conditions, as the network size increases from small to large, the number of enterprises choosing the IDT strategy for digital transformation gradually decreases, while the number of enterprises adopting the PDT strategy increases. Additionally, when the network size exceeds a certain threshold, enterprises stop choosing digital transformation altogether.

This outcome can be attributed to the fact that, with a smaller network size, each enterprise has a larger average market demand, leading to greater market share gains from digital transformation. In such cases, the IDT strategy significantly reduces production costs. Thus, with smaller network sizes, more enterprises opt for the IDT strategy for digital transformation. However, as the network size grows, the average market demand for each enterprise decreases, and the market share gained from digital transformation also diminishes. The effectiveness of the IDT strategy in reducing production costs weakens and fails to cover the high costs of digital transformation, leading to a gradual increase in the number of enterprises choosing the PDT strategy. When the network size further expands, the market share gained through digital transformation becomes increasingly negligible. Regardless of the digital strategy adopted, the returns do not cover the investment costs, ultimately leading all enterprises to abandon digital transformation in favor of the STM strategy. This phenomenon indicates that network size has a significant impact on the choice of digital transformation strategies, and there is a clear scale effect. Different network sizes require digital platforms to implement tailored pricing strategies and service quality to effectively promote enterprise digital transformation.

#### 4.3.3. Level of Irrationality

**Figure 9:**
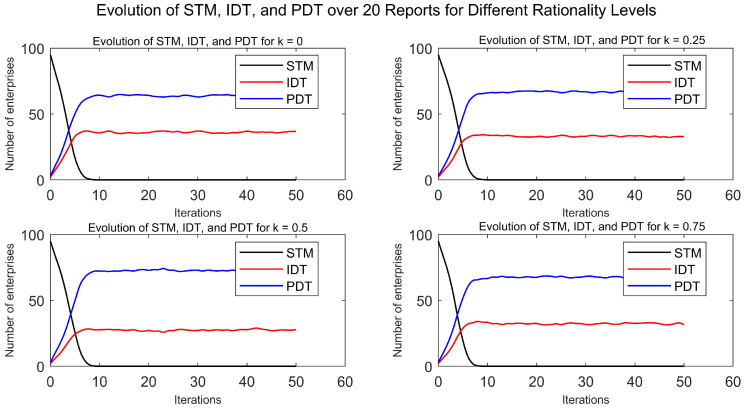
Enterprise digital transformation under different levels of rationality.

To examine the impact of the learning rule parameter k on the conclusions, simulations were conducted with values of 0, 0.25, 0.5, and 0.75. The results are shown in Figure 9. A vertical comparison reveals that, as the level of irrationality increases, the number of enterprises choosing the PDT strategy for digital transformation first increases and then decreases.

This phenomenon may stem from the rationality level of enterprise decisions. Under conditions of complete rationality, enterprises will tend to imitate strategies that exhibit higher returns, even in the face of small differences in returns, to maximize their profits. Therefore, the distribution of enterprises choosing IDT or PDT strategies is the result of a fully rational analysis. As irrational factors increase in enterprise decision-making, the sensitivity to return differences decreases. This reduced sensitivity leads enterprises to base their strategy choices less strictly on cost-effectiveness, potentially influenced by other non-economic factors, reflected in the increased number of PDT strategy adopters. However, as the level of irrationality intensifies further, the decision-making process becomes more uncertain and random. In such cases, the number of enterprises choosing the PDT strategy may decrease, potentially returning to levels similar to those under complete rationality. This reversion may be due to the inability of enterprises to continuously identify and imitate high-return strategies in highly irrational decision-making, leading to increased randomness in strategy choices and thus balancing the distribution of enterprises between the two strategies.

#### 4.3.4. Preference Learning Rules

Within the framework of the Fermi–Dirac distribution, enterprises are traditionally considered to randomly select a competitor as their learning target when updating strategies. However, in real business environments, enterprises often exhibit a degree of selectivity when choosing their learning targets. This selection is not entirely random but is influenced by factors such as the strength and social status of the competitor being observed.

To test the robustness of this phenomenon, this study adopts a preferential attachment learning rule to replace the traditional random matching mechanism and applies the Fermi–Dirac rule for numerical simulation analysis. The mathematical expression for the preferential attachment learning rule is as follows:

$$P_{\left(s_{i}\to s_{j}\right)}=\frac{d_{j}^{\vartheta }}{\sum_{k\in N:(k,l)\in L}d_{k}^{\vartheta }},$$

where $d_{j}$ represents the degree of competitor j (i.e., its influence or number of connections in the network), $\sum_{k\in N:\left(k,l\right)\in L}d_{k}$ is the sum of degrees of all competitors of enterprise i, and ϑ is an adjustable parameter. The higher the value of ϑ, the greater the probability that enterprise i will choose a competitor with a higher influence as its learning target. In this setting, ϑ is set to 1, meaning that the learning probability is proportional to the competitor’s influence.

The core idea of this rule is that, after each round of the game, enterprise i selects a competitor for strategy learning based on the probability distribution $P_{\left(s_{i}\to s_{j}\right)}$, where the influence of the competitor is considered as its node degree in the network. This influence-based selection mechanism more closely aligns with rational behavior in real-world business decision-making processes, providing deeper insights into strategy learning and evolutionary dynamics of enterprises in complex network environments.

**Figure 10:**
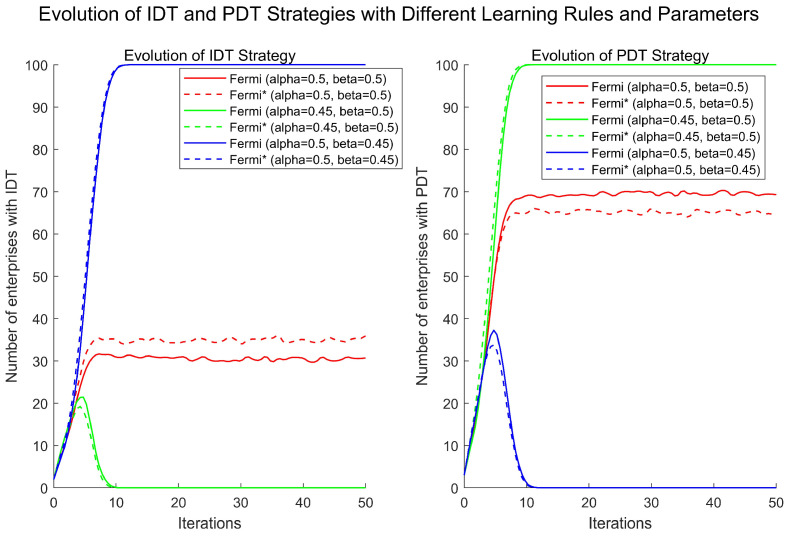
Comparison of the modified Fermi rule. Feimi* indicates the modified strategy update rule.

The simulation results are shown in Figure 10, revealing that, under the modified strategy update rules, the impact of digital platform service pricing and service quality on enterprise digital transformation strategy choices remains significant. Comparative analysis shows that, when there is a significant difference in cost-effectiveness between IDT and PDT strategies, the impact of the modified strategy on the direction of enterprise digital transformation is minimal, mainly accelerating the convergence speed of the system’s steady state. However, when the cost-effectiveness of the two strategies is similar and it is difficult to distinguish between them, the modified strategy leads to a decrease in the number of enterprises choosing the PDT strategy.

This phenomenon can be explained by the fact that, when there is a significant difference in cost-effectiveness between IDT and PDT strategies, enterprises tend to choose the more cost-effective strategy. Even with the introduction of the modified strategy update rules, the overall choice trend remains fundamentally unchanged. Conversely, when the cost-effectiveness difference between the two strategies is minimal, enterprises become more cautious in their decision-making, and the modified strategy reduces the preference for the PDT strategy. This indicates that the modified strategy update rules increase enterprises’ sensitivity to subtle differences in cost-effectiveness, thereby influencing their final choices.

#### 4.3.5. Comparative Analysis

In the real world, a company’s decision to undergo digital transformation is influenced by multiple factors, including product prices, production costs, market demand, digital investment costs, and the effectiveness of digitalization. The varying combinations of these factors can lead to changes in the impact and effectiveness of digital platform pricing and service quality on enterprise transformation strategies. To investigate these specific effects and validate the robustness of the research findings, this study adjusts key parameters such as market demand, product prices, digital investment costs, and digital effectiveness. Specifically, the study computes and compares the results of enterprise digital transformation under the conditions of α=0.5,β=0.5 and α=0.55,β=0.55.

The results are shown in Table 3, where the final column indicates the number of enterprises choosing STM, IDT, and PDT strategies. Comparing the steady-state enterprise proportions under the initial conditions reveals that, when the market demand is high, product prices are low, or when digital effectiveness is high, enterprises are more likely to choose the STM strategy for independent digital transformation. This choice allows for the high adaptability of digital content, which helps to meet market demand while further reducing production costs. When digital investment costs are high, enterprises tend to rely on the PDT strategy, utilizing digital platforms to achieve digital transformation with lower investment costs. Additionally, when digital effectiveness is low (i.e., digital transformation does not significantly reduce production costs), enterprises typically do not choose to undertake digital transformation.

### 4.4. Results Analysis

This study examines how the pricing and service quality of digital platforms influence corporate strategic choices in digital transformation. The results demonstrate that these factors significantly shape enterprise decision-making, with their effects mediated by information flow, bounded rationality, and information asymmetry.

When the service quality is constant, lower platform pricing encourages enterprises to adopt the platform-driven transformation (PDT) strategy, while higher pricing increases the proportion of firms opting for independent digital transformation (IDT). High-cost-efficiency platform services lead enterprises to choose PDT strategies, optimizing decision-making and mitigating uncertainty. Conversely, when the cost-effectiveness of platform services is inferior to that of independent digital initiatives, firms are more likely to adopt the IDT approach. Additionally, decisions regarding digital transformation are also influenced by factors such as the level of enterprise rationality, learning preferences, market demand, product pricing, digital effectiveness, and digital costs. Based on the research results, the following insights were further identified:

(1) Stabilizing decision-making through improved platform services. Digital transformation provides opportunities for cost reduction and market expansion but entails substantial upfront investments and uncertain returns. In low-demand markets, enterprises may refrain from transformation if costs exceed the potential benefits. Digital platforms, by enhancing service quality and reducing costs, can mitigate system uncertainty, enabling more rational decision-making in complex environments. Lowering platform costs is particularly crucial in such scenarios, as it alleviates financial constraints, reduces information asymmetry, and increases the likelihood of successful digital transformation.

(2) Optimizing service pricing and quality to reduce uncertainty. Service pricing and quality directly influence both strategic decisions and system uncertainty, reflected in entropy changes. In industries with pronounced information asymmetry, excessive pricing or inadequate quality may prevent enterprises from achieving transformation benefits, exacerbating uncertainty and entropy. In contrast, high-value platform services encourage firms to pursue PDT strategies, optimizing decision-making processes and minimizing entropy increases. Effective pricing and quality strategies not only enhance the competitiveness but also reduce decision-making entropy, thereby accelerating transformation progress.

(3) Driving sustainable competitive advantage through digital transformation. Digital transformation is pivotal for achieving sustainable competitive advantages in competitive markets. Early adopters leverage lean production and market insights to reduce costs and expand demand, lowering system entropy and uncertainty. As more firms engage in digital transformation, competition intensifies and entropy effects become more pronounced. Enterprises that improve profitability through reduced costs and reap transformation benefits that outweigh expenses contribute to a self-reinforcing transformation process, propelling industry-wide digitalization. This interplay of information flow and entropy dynamics underscores the transformative impact of digital adoption.

(4) Enhancing societal productivity through digital transformation. Digital transformation transcends individual enterprise productivity, fostering broader societal productivity improvements. Initially, pioneering firms capture surplus market demand, but as participation grows, the surplus demand diminishes, and the information flow within the system becomes more ordered, stabilizing entropy over time. This progression highlights digital transformation’s role as a key driver of systemic productivity enhancement. Ultimately, its core value lies in reducing costs through improved digital efficiency and achieving sustainable growth via effective information dissemination and resource optimization.

## 5. Conclusions and Recommendations

### 5.1. Conclusions

This study employs complex network evolutionary game theory to construct a game model for enterprise digital transformation. It extends the application of the Fermi–Dirac rule in decision-making under uncertainty and highlights the role of information entropy in enterprise strategic choices, offering a new perspective on the impact of bounded rationality in digital ecosystems. These findings enrich the theoretical discussion on platform pricing, service quality, and enterprise adaptation, contributing to the literature on strategic decision-making and digital transformation. Firstly, service pricing and quality are pivotal in shaping enterprise digital transformation. These factors play a critical role in promoting transformation and ensuring the sustainable development of digital platforms. Influenced by social networks, pricing and quality directly affect the efficiency of information dissemination, which, in turn, drives the evolution of enterprise decision-making.

Secondly, in contexts characterized by low market demand, low digitalization efficiency, and extensive network scales, digital platforms should optimize information flow by reducing service pricing and enhancing quality, thereby effectively advancing enterprise digital transformation.

Thirdly, enterprise rationality levels significantly influence transformation strategy selection. Both excessive rationality and irrational tendencies hinder optimal decision-making. The entropy increase observed during this process is closely tied to decision evolution, with information asymmetry and bounded rationality serving as critical contributors to decision uncertainty.

Lastly, optimizing digital platforms regulates information flow and reduces system entropy. This not only influences enterprise decision-making but also enables more rational and sustainable choices in complex and uncertain market environments.

### 5.2. Recommendations

The conclusions of this study provide the following four policy implications:

(1) Expand investment in foundational digital platform infrastructure. Governments should focus on advancing critical technologies such as cloud computing, big data analytics, and artificial intelligence. Establishing open data-sharing platforms would enhance information flow, mitigate asymmetry, and provide a robust technical foundation for enterprise digital transformation.

(2) Implement differentiated service strategies tailored to diverse enterprise needs. Customizing service packages based on firm size, industry, and maturity can optimize service quality and pricing, reduce decision-making uncertainty, and lower system entropy, ensuring smoother transformation processes.

(3) Provide decision-making training and consulting services. Training programs can equip decision-makers with the tools to analyze market trends, evaluate cost–benefit trade-offs, and manage risks, addressing the decision-making challenges posed by bounded rationality and information asymmetry.

(4) Offer fiscal incentives to encourage high-value platform services. By lowering costs, improving quality, and increasing service value, digital platforms can optimize information flow, reduce entropy, and alleviate enterprise financial pressures during transformation.

### 5.3. Weaknesses and Prospects

While the study offers valuable insights into the role of digital platforms in enterprise digital transformation, further exploration is warranted:

1. Refining the Fermi–Dirac rule to include social and psychological factors such as cultural norms and emotional influences, enabling a more comprehensive reflection of enterprise decision-making in complex environments.
2. Enhancing the model by incorporating dynamic variables such as market demand fluctuations and technological advancement rates, enabling a more nuanced evaluation of risks and benefits while addressing entropy within information flows.
3. Incorporating collaborative dynamics into enterprise interactions, broadening the scope beyond competition to more accurately depict decision-making in complex network environments.
4. Validating the model through empirical studies, providing stronger support for simulation outcomes and offering deeper theoretical insights into the interplay between information flow and decision-making uncertainty.

## Figures and Tables

**Table 1:** Initial parameter values for the simulation.

Parameter	N	P	Q (10,000)	C	I (10,000)	$C^{DT}$	β	α	θ	ω	γ
Initial Value	100	120	250	100	55	80	0.5	0.5	0.1	5	0.05

**Table 2:** Average revenue of the enterprises in digital transformation under different parameter combinations.

Parameter Combinations	$\bar{U_{IDT}}$ (10,000)	$\bar{U_{PDT}}$ (100,000)
α=0.4, β=0.5	0	60.1
α=0.45, β=0.5	0	57.4
α=0.5, β=0.5	54.6	54.6
α=0.55, β=0.5	54.5	2.6
α=0.6, β=0.5	54.5	0
α=0.5, β=0.43	54.5	0
α=0.5, β=0.46	54.5	28.5
α=0.5, β=0.49	54.6	53.2
α=0.5, β=0.52	46.2	55.8
α=0.5, β=0.55	0	57.4

**Table 3:** Parameter sensitivity analysis.

Benchmark Parameters	Change Rate	Change Parameters	Number of Enterprises Choosing Different Strategies	
			α=0.5, β=0.5;	α=0.55, β=0.55
Initial	0	-	0, 37, 63	0, 38, 62
Q (10,000)	10%	275	0, 100, 0	0, 100, 0
	−10%	225	0, 0, 100	0, 0, 100
P	10%	132	0, 33, 67	0, 38, 62
	−10%	108	0, 84, 16	0, 85, 15
I (10,000)	10%	60.5	0, 0, 100	0, 0, 100
	−10%	49.5	0, 100, 0	0, 100, 0
$C^{DT}$	10%	88	100, 0, 0	100, 0, 0
	−10%	72	0, 100, 0	0, 100, 0

## Data Availability

The original contributions presented in the study are included in the article, and further inquiries can be directed to the corresponding authors.

## References

[1] Bhimani A. (2015). Exploring Big Data’s Strategic Consequences. J. Inf. Technol..

[2] Majchrzak A., Markus M.L., Wareham J. (2016). Designing for digital transformation. MIS Q..

[3] Morakanyane R.G., Audrey A., O’Reilly P. (2017). Conceptualizing Digital Transformation in Business Organizations: A Systematic Review of Literature. BLED 2017 Proceedings.

[4] (2015). United Nations, Department of Economic and Social Affairs. Transforming Our World: The 2030 Agenda for Sustainable Development.

[5] Verhoef P.C., Broekhuizen T., Bart Y., Bhattacharya A., Dong J.Q., Fabian N., Haenlein M. (2021). Digital transformation: A multidisciplinary reflection and research agenda. J. Bus. Res..

[6] Chinese Academy of Cyberspace Studies (2024). World Internet Development Report 2022.

[7] Quinton S., Canhoto A., Molinillo S., Pera R., Budhathoki T. (2018). Conceptualising a digital orientation: Antecedents of supporting SME performance in the digital economy. J. Strateg. Mark..

[8] Podolny J.M., Stuart T.E., Hannan M.T. (1996). Networks, Knowledge, and Niches: Competition in the Worldwide Semiconductor Industry, 1984–1991. Am. J. Sociol..

[9] Chen C.-L., Lin Y.-C., Chen W.-H., Chao C.-F., Pandia H. (2021). Role of Government to Enhance Digital Transformation in Small Service Business. Sustainability.

[10] Doblinger C., Surana K., Anadon L.D. (2019). Governments as partners: The role of alliances in U.S. cleantech startup innovation. Res. Policy.

[11] Cenamor J., Parida V., Wincent J. (2019). How entrepreneurial SMEs compete through digital platforms: The roles of digital platform capability, network capability and ambidexterity. J. Bus. Res..

[12] Schönfuß B., McFarlane D., Hawkridge G., Salter L., Athanassopoulou N., de Silva L. (2021). A catalogue of digital solution areas for prioritising the needs of manufacturing SMEs. Comput. Ind..

[13] McIntyre D.P., Srinivasan A. (2017). Networks, platforms, and strategy: Emerging views and next steps. Strateg. Manag. J..

[14] Li L., Su F., Zhang W., Mao J.-Y. (2018). Digital transformation by SME entrepreneurs: A capability perspective. Inf. Syst. J..

[15] Ben Mahmoud-Jouini S., Lenfle S. (2010). Platform re-use lessons from the automotive industry. Int. J. Oper. Prod. Manag..

[16] Lee S.M., Kim T., Noh Y., Lee B. (2010). Success factors of platform leadership in web 2.0 service business. Serv. Bus..

[17] Industrial Internet Consortium (2019). The Industrial Internet Reference Architecture v1.9.

[18] Lee H.-H., Kwon J.-H., Kim E.-J. (2018). FS-IIoTSim: A flexible and scalable simulation framework for performance evaluation of industrial Internet of things systems. J. Supercomput..

[19] Liu C., Su Z., Xu X., Lu Y. (2022). Service-oriented industrial internet of things gateway for cloud manufacturing. Robot. Comput.-Integr. Manuf..

[20] Bian L., Zhang J., Cui Q., Chen X., Wang S. (2021). Research on the Realization and Application of Intelligent IoT Platform for Electrical Equipment under Industrial Internet. J. Phys. Conf. Ser..

[21] Zhang X., Ming X. (2021). A comprehensive industrial practice for Industrial Internet Platform (IIP): General model, reference architecture, and industrial verification. Comput. Ind. Eng..

[22] Parida V., Sjödin D., Reim W. (2019). Reviewing Literature on Digitalization, Business Model Innovation, and Sustainable Industry: Past Achievements and Future Promises. Sustainability.

[23] Guo S., Zhang P., Yang J. (2018). System dynamics model based on evolutionary game theory for quality supervision among construction stakeholders. J. Civ. Eng. Manag..

[24] Young H.P. (2020). Individual Strategy and Social Structure: An Evolutionary Theory of Institutions.

[25] Li J., Shang Y., Wang W., Ma J. (2024). Bipartite consensus of concatenated opinion dynamics for two antagonistic groups: A game theoretical perspective. Neurocomputing.

[26] Zhang W., Zhao S., Wan X. (2021). Industrial digital transformation strategies based on differential games. Appl. Math. Model..

[27] Filieri R., Alguezaui S. (2014). Structural social capital and innovation. Is knowledge transfer the missing link?. J. Knowl. Manag..

[28] Granovetter M. (1985). Economic Action and Social Structure: The Problem of Embeddedness. Am. J. Sociol..

[29] Hansen M.T. (1999). The Search-Transfer Problem: The Role of Weak Ties in Sharing Knowledge across Organization Subunits. Adm. Sci. Q..

[30] Wu B., Liu P., Xu X. (2017). An evolutionary analysis of low-carbon strategies based on the government–enterprise game in the complex network context. J. Clean. Prod..

[31] Ellström D., Holtström J., Berg E., Josefsson C. (2022). Dynamic capabilities for digital transformation. J. Strategy Manag..

[32] Yousaf Z. (2021). Go for green: Green innovation through green dynamic capabilities: Accessing the mediating role of green practices and green value co-creation. Environ. Sci. Pollut. Res..

[33] Yang Y., Chen N., Chen H. (2023). The Digital Platform, Enterprise Digital Transformation, and Enterprise Performance of Cross-Border E-Commerce—From the Perspective of Digital Transformation and Data Elements. J. Theor. Appl. Electron. Commer. Res..

[34] Yoo Y., Henfridsson O., Lyytinen K. (2010). Research Commentary—The New Organizing Logic of Digital Innovation: An Agenda for Information Systems Research. Inf. Syst. Res..

[35] Parker G.G., Van Alstyne M.W., Choudary S.P. (2016). Platform Revolution: How Networked Markets Are Transforming the Economy and How to Make Them Work for You.

[36] Saarikko T., Westergren U.H., Blomquist T. (2020). Digital transformation: Five recommendations for the digitally conscious firm. Bus. Horiz..

[37] Elia G., Margherita A., Passiante G. (2020). Digital entrepreneurship ecosystem: How digital technologies and collective intelligence are reshaping the entrepreneurial process. Technol. Forecast. Soc. Change.

[38] Dalenogare L.S., Benitez G.B., Ayala N.F., Frank A.G. (2018). The expected contribution of Industry 4.0 technologies for industrial performance. Int. J. Prod. Econ..

[39] Xu S.Z., Da B. (2013). Complex Network Model and Its Application. Adv. Mater. Res..

[40] Cheng D., He F., Qi H., Xu T. (2015). Modeling, Analysis and Control of Networked Evolutionary Games. IEEE Trans. Autom. Control.

[41] He S., Tang Y. (2023). Effects of Personalized Demands on the Digital Diffusion of Enterprises: A Complex Network Evolution Game Model-Based Study. J. Knowl. Econ..

